# Comparison of Genomic and Epigenomic Expression in Monozygotic Twins Discordant for Rett Syndrome

**DOI:** 10.1371/journal.pone.0066729

**Published:** 2013-06-21

**Authors:** Miyake Kunio, Chunshu Yang, Yohei Minakuchi, Kenta Ohori, Masaki Soutome, Takae Hirasawa, Yasuhiro Kazuki, Noboru Adachi, Seiko Suzuki, Masayuki Itoh, Yu-ichi Goto, Tomoko Andoh, Hiroshi Kurosawa, Wado Akamatsu, Manabu Ohyama, Hideyuki Okano, Mitsuo Oshimura, Masayuki Sasaki, Atsushi Toyoda, Takeo Kubota

**Affiliations:** 1 Department of Epigenetic Medicine, Faculty of Medicine, Interdisciplinary Graduate School of Medicine and Engineering, University of Yamanashi, Chuo, Japan; 2 Comparative Genomics Laboratory, Center for Information Biology, National Institute of Genetics, Mishima, Japan; 3 Department of Biomedical Science, Institute of Regenerative Medicine and Biofunction, Graduate School of Medical Science, Tottori University, Yonago, Japan; 4 Department of Legal Medicine, Interdisciplinary Graduate School of Medicine and Engineering, University of Yamanashi, Chuo, Japan; 5 Department of Child Neurology, National Center Hospital for Mental, Nervous, and Muscular Disorders, National Center of Neurology and Psychiatry, Tokyo, Japan; 6 Department of Mental Retardation and Birth Defect Research, National Institute of Neuroscience, National Center of Neurology and Psychiatry, Tokyo, Japan; 7 Department of Biotechnology, Faculty of Life and Environmental Sciences, Interdisciplinary Graduate School of Medicine and Engineering, University of Yamanashi, Kofu, Japan; 8 Department of Physiology, Keio University School of Medicine, Keio University School of Medicine, Tokyo, Japan; 9 Department of Dermatology, Keio University School of Medicine, Keio University School of Medicine, Tokyo, Japan; University of Insubria, Italy

## Abstract

Monozygotic (identical) twins have been widely used in genetic studies to determine the relative contributions of heredity and the environment in human diseases. Discordance in disease manifestation between affected monozygotic twins has been attributed to either environmental factors or different patterns of X chromosome inactivation (XCI). However, recent studies have identified genetic and epigenetic differences between monozygotic twins, thereby challenging the accepted experimental model for distinguishing the effects of nature and nurture. Here, we report the genomic and epigenomic sequences in skin fibroblasts of a discordant monozygotic twin pair with Rett syndrome, an X-linked neurodevelopmental disorder characterized by autistic features, epileptic seizures, gait ataxia and stereotypical hand movements. The twins shared the same *de novo* mutation in exon 4 of the *MECP2* gene (G269AfsX288), which was paternal in origin and occurred during spermatogenesis. The XCI patterns in the twins did not differ in lymphocytes, skin fibroblasts, and hair cells (which originate from ectoderm as does neuronal tissue). No reproducible differences were detected between the twins in single nucleotide polymorphisms (SNPs), insertion-deletion polymorphisms (indels), or copy number variations. Differences in DNA methylation between the twins were detected in fibroblasts in the upstream regions of genes involved in brain function and skeletal tissues such as *Mohawk Homeobox* (*MKX*), *Brain-type Creatine Kinase* (*CKB*), and *FYN Tyrosine Kinase Protooncogene* (*FYN*). The level of methylation in these upstream regions was inversely correlated with the level of gene expression. Thus, differences in DNA methylation patterns likely underlie the discordance in Rett phenotypes between the twins.

## Introduction

Rett syndrome (RTT) is an X-linked dominant neurodevelopmental disorder that predominantly affects females with an incidence of one in 10,000–15,000 female births [1], [2]. Affected patients manifest various neuro-psychiatric features, including autistic features, epileptic seizures, gait ataxia, and stereotypical hand movements [1]. Mutation of the *MECP2* gene (Xq28) accounts for approximately 90% of cases with classic RTT [3].

Twins with clinically well-characterized RTT have been identified [4]–[10]. To date, *MECP2* mutations have been confirmed in only two cases, a point mutation (R294X) and a deletion in exon 3, respectively [9], [10]. Phenotypic differences between the twins has been attributed to differences in their X chromosome inactivation (XCI) patterns, with skewing in favor of the paternal allele (the presumptive mutated allele) in the twin with the more severe clinical phenotype [9].

If the concordance rate between monozygotic (MZ) twins approaches 100%, then the trait under study is likely to be under genetic control with high penetrance. For some traits, however, MZ concordance rates are much lower than 100%, indicating a role for environmental and/or stochastic epigenetic factors in modifying the phenotype associated with the underlying genotype. Epigenetic mechanisms are widely believed to mediate the effect of environmental factors on the genome and, as a result, the use of twins in epigenetic research is becoming increasingly popular [11], [12]. Comparative analysis of the epigenome has recently been conducted in twins showing discordances in mental disease [13], [14], multiple sclerosis [15], or without any specific disease [16]. Some of these studies examined DNA methylation at specific imprinted loci [17]–[20], whereas, genome-wide DNA methylation was analyzed in others [14]–[16], [21]. In this report, we compared whole genome sequences and DNA methylation patterns in discordant RTT twins with a *MECP2* mutation in order to determine the genetic or epigenetic factor(s) responsible for the inter-twin differences in clinical severity. To our knowledge, this is the first study to make use of this approach in discordant twins with a disease with a disease (Rett syndrome) involving failure of an epigenetic mechanism.

## Materials and Methods

### Patients’ Samples

Peripheral blood leukocytes and skin fibroblasts were obtained from the twin patients. Written informed consent was obtained from parents of the twins before collection of the patient samples and prior to publishing details of the patient cases. The study protocol was reviewed and approved by the research ethics committees of University of Yamanashi (Nos. 523, 699), National Institute of Genetics (Nos. 22-1, 23-19), and Keio University (No. 20080016). This study was conducted in accordance with the principles expressed in the Declaration of Helsinki.

### DNA Sequencing of the *MECP2* Gene

DNA was extracted from peripheral blood leukocytes and skin fibroblasts according to standard procedures. PCR was performed using seven primers designed to cover the entire *MECP2* coding region as described previously (Amir et al. 1999). The PCR product was purified using a commercial PCR purification kit (Qiagen, Valencia, CA, USA) and bidirectional sequenced using an ABI Prism 310 sequencer (Applied Biosystems, Foster City, CA, USA). Sequence data of the subjects were compared to the published reference genomic sequences (RefSeqGene on chromosome X NCBI Reference Sequence: NG_007107.1).

### Generating Mouse Hybrid Cells Containing a Single Patient-derived X-chromosome

The construction of mouse A9 cells containing a single human X chromosome was carried out as reported previously [22]. Briefly, mouse A9 cells were fused with recipient cells for the introduction of a single human chromosome X. The parental origin of a single patient-derived human chromosome X in each hybrid clone was determined by polymorphic analysis.

### Bisulfite Treatment

Genomic DNA was extracted using a DNeasy Blood and Tissue Kit (Qiagen, Hilden, Germany), and was subjected to sodium bisulfite modification with an EZ DNA Methylation-Gold kit (Zymo Research, CA, USA) as described previously [23].

### X-chromosome Inactivation Analyses

The human androgen receptor gene locus (HUMARA) assay using methylation-specific PCR was performed as described previously [24]. Briefly, the assay uses a bisulfite-treatment followed by PCR; the inactive X pattern is based on the methylated allele at the HUMARA locus and the active X pattern is based on the unmethylated allele at the same locus. Both patterns were used to calculate the XCI ratio.

### Whole-genome Sequencing

High-quality genomic DNA from the twins were fragmented using a Covaris Acoustic Solubilizer S220. A paired-end library from each sheared DNA was prepared using the Illumina Paired End DNA Sample Prep kit according to the manufacturer’s protocol. The concentration and size distribution of the libraries were quantified using an Agilent 2100 Bioanalyzer. The libraries were run on the Illumina HiSeq2000 sequencers and read length was 100 bp.

### Mapping and Variant Calling

Paired-end reads were aligned to the human reference sequence (NCBI Build 36.1) using the Burrows-Wheeler Aligner program [25], and duplicated reads were removed using the Picard program. Variant calling from the pileup file obtained by the SAMtools program [26] was performed using the following filter: (i) paired-end reads were aligned uniquely for at least two reads of each orientation, (ii) mapped read depth for variant calling was 20x or more coverage, (iii) mismatches with a base quality score of less than 13 were discarded. Variants with a read ratio ≥0.3, which is defined as the ratio of variant read depth to total read depth at each position, were genotyped as heterozygous variants and those with a ratio ≥0.8 were genotyped as homozygous variants. The BreakdancerMax-1.1 program [27] was used to detect the structural variants for 10x or more coverage. To detect copy number variants (CNVs), short reads were mapped to the human reference genome, which was masked by RepeatMasker and TRF programs, using mrFAST (v2.0.0.5) and allowing up to 5% mismatches. Then, copy number over 5 kb was predicted by mrCaNaVaR (v0.3).

### BeadChip Array Analysis

SNP genotyping and CNV detection were performed using the Illumina Infinium HD Assay system (HumanOmni2.5-Quad). Genomic DNA from the twins were tested on duplicated arrays. Data analysis for SNPs and CNV probes was performed using the Illumina’s GenomeStudio software.

### Digital Droplet PCR Assay

The digital droplet PCR assay was performed as described previously [28], [29]. Briefly, target primer pairs were designed using ProbeFinder software from Roche Applied Science. The Droplet Digital PCR workflow begins by partitioning the universal probe library (UPL) probe (Roche, Tokyo, Japan) reaction mix containing DNA into aqueous droplets in oil via the QX100 Droplet Generator; after transfer of droplets to a 96-well PCR plate, a 2-step thermocycling protocol (95°C × 10 min; 40 cycles of [94°C × 30 s, 60°C × 60 s (ramp rate set to 2°C/s)], 98°C × 10 min) is carried out in a conventional thermal cycler, and the PCR plate is then transferred to the QX100 Droplet Reader (a droplet flow cytometer) for automatic reading of samples in all wells. Bio-Rad QX100 reagents and consumables were used for the experiments including droplet generator oil (186–3005), DG8™ cartridges and gaskets (186–3006), droplet reader oil (186–3004), and ddPCR supermix for probes (186–3010). UPL probe assays were used at a final concentration of 1× (1 µM of each forward and reverse target primer; 2 µM of each RNaseP forward and reverse primer; 250 nM of each FAM UPL probe) in all ddPCR reactions. All reactions for each primer set were run in triplicate.

### Bisulfite Sequencing

Bisulfite modified DNA was amplified by PCR with the primers *MECP2*-BF1 (5′-GTTAGGTTTTAGGGTGGGTAATTTT-3′) and *MECP2*-BR1 (5′-CCCCTCCAACTATTAATTAACTACTTTC-3′), *MECP2*-BF2 (5′- GAGGTTTTGGTATGTATTTTTTTT-3′) and *MECP2*-BR2 (5′- ATTACCCACCCTAAAACCTAAC- 3′) specific to the *MECP2*- promoter region, *MKX*-BF (5′-TTAGGGTTGTAGGTGTAAAA-3′) and *MKX*-BR (5′-TACTATTAACCCCTAACAAAAAAAC-3′) specific to the *MKX*- upstream region, *CKB*-BF (5′-GATTTGTTTAAGGTTAGGGTAT-3′) and *CKB*BR (5′-CCTCAAATCCTTAAATATCTAAATCCC-3′) specific to the *CKB*-upstream region and, *FYN-*BF (5′-AATTTTTAAAAATATATAGATTTTTTTT- 3′) and *FYN-*BR (5′-TATCAAAAAACCCCTTAAAAACTAC-3′) specific to the *FYN*-upstream region using one cycle of 95°C for 10 min, 35 cycles of 95°C for 30 s, 55°C for 30 s, 72°C for 30 s, with a final cycle of 72°C for 7 min. Each PCR product was cloned into a pTAC-2 vector using a TA PCR Cloning Kit (BioDynamics, Tokyo, Japan) and sequenced.

### Quantitative Reverse Transcription PCR Assay (qRT-PCR)

Total RNA was reverse-transcribed with random primers and reverse transcriptase (TOYOBO, Osaka, Japan) according to the manufacturer’s instructions. One tenth of the reaction was used in the PCR amplification. Gene expression was measured by qRT-PCR on an ABI Prism 7500 with a THUNDERBIRD SYBR Green PCR mix (TOYOBO) using primers *MECP2* (QT00065933, Qiagen), *MKX*-F (5′-ATCGCACAGACACTCTGGAA-3′) and *MKX-R* (5′-CCATAGCTGCGTTGATCTCCT-3′), *CKB*-F (5′-GCCTCACCCAGATTGAAACTCTCT-3′) and *CKB-R* (5′-GGTTGGGCAGCTTGATATGCAC-3′), *FYN*-F (5′-TCAAGTCTGACGTGTGGTCTT-3′) and *FYN-R* (5′-CTCCCGGTTGTTCATGCCT-3′). The expression level of each gene was normalized against that of human *GAPDH*. All qRT-PCRs were performed three times using duplicated samples. The Mann-Whitney U test was performed to compare the relative expression levels between twins.

### Array-based Genome-wide DNA Methylation Analysis

Array-based genome-wide DNA methylation analysis was performed using the Infinium HumanMethylation450 BeadChip (Illumina) according to the manufacturer’s instructions. GenomeStudio normalizes data using different internal controls that are present on the HumanMethylation450 BeadChip. It also normalizes data depending on internal background probes. The methylation status of specific cytosines is indicated by an average (AVG) beta value where 1 corresponds to complete methylation and 0 to no methylation. Signals of probes with *P*≥0.05 were excluded from the analysis. The comparison of methylation patterns between the twins was based on the difference in (AVG) beta value of each CpG site. DiffScore, a value obtained by an Illumina software (GenomeStudio Methylation module, ver.1.9), is used for illustration of the difference of two groups of data. For a locus covered by multiple probes, the DiffScores across probes were averaged (DiffScore <|374.344|; control = 0). An absolute value >80 was considered to indicate differentially methylated CpG sites between the twins. Methylation microarray data were deposited in the GEO database (accession number GSE43141).

## Results

### Probands

The twins were delivered spontaneously at 37 weeks of gestation to healthy, non-consanguineous parents, when both parents were 25 years old. Their 16-year-old brother was healthy. Twin 1 (RS1), who has a milder phenotype, developed apparently normally until 2.5 years of age; she was able to use a spoon, to run and jump, and to climb stairs. At age 2, she communicated using two-word sentences. At 2.5 years, she started to lose learned words and the ability to communicate. At 3 years and 4 months, her EEG was abnormal, but she did not have seizures. At 3 years and 5 months, she lost purposeful hand skills and started to exhibit stereotypical hand movements. At 3.5 years, she weighed 12 kg (−1.6 SD), measured 91 cm (−1.1 SD), and had an occipito-frontal circumference (OFC) of 47.5 cm (−0.7 SD). At age 12 years, she had generalized convulsions and her EEG showed epileptic discharges. Since then, an antiepileptic drug has been administered and her seizures are currently well-controlled. At age 13 years, she could run and jump, was rather hyperactive and thus slimmer than twin 2, was able to reach for and grasp desired objects, liked to swim and to watch children’s TV programs.

Twin 2 (RS2), who has a more severe phenotype, had an OFC at birth of 32.3 cm (−0.6 SD). Her development during the first 6 months appeared normal but she soon started to lag. She was able to hold her head steady at 6 months, could roll over at 9 months, and could sit up by herself at 9 months. She had marked hypotonia and never walked. At age 12 months, she spoke using simple words, such as “momma” and “dada,” and could grasp a toy, but she lost these abilities later and started to exhibit stereotypical hand movements. At age 3.5 years, her weight was 11.34 kg (−2.0 SD), her length, 94.9 cm (−2.1 SD), and her OFC, 45.3 cm (−2.1 SD). At 2 years and one month, she had afebrile seizures, and her EEG showed epileptiform spike discharges. At 6 years of age, an antiepileptic drug was administered. At age 7 years, she was unable to stand, walk, speak or communicate with others. At age 13 years, she could not stand and required a wheelchair.

The clinical score used to grade the severity of RTT [30] was 7 for RS1 (milder) and 27 for RS2 (more severe). Based on more recent diagnostic criteria for RTT, RS2 showed the most important criterion (a period of regression) and fulfilled all other main criteria (loss of acquired purposeful hand skills, loss of acquired spoken language, gait abnormalities and stereotypical hand movements) indicating that she has “typical RTT”; by contrast, although RS1 showed the most important criterion (a period of regression), she only fulfilled two of the four main criteria (loss of acquired spoken language and stereotypical hand movements) indicating that she has “atypical RTT” [31]. Twenty-one informative microsatellite markers [32] were genotyped in the twins, and identical alleles were observed at all markers confirming their monozygosity.

### Mutation Detection of *MECP2* Gene

**Figure 1:**
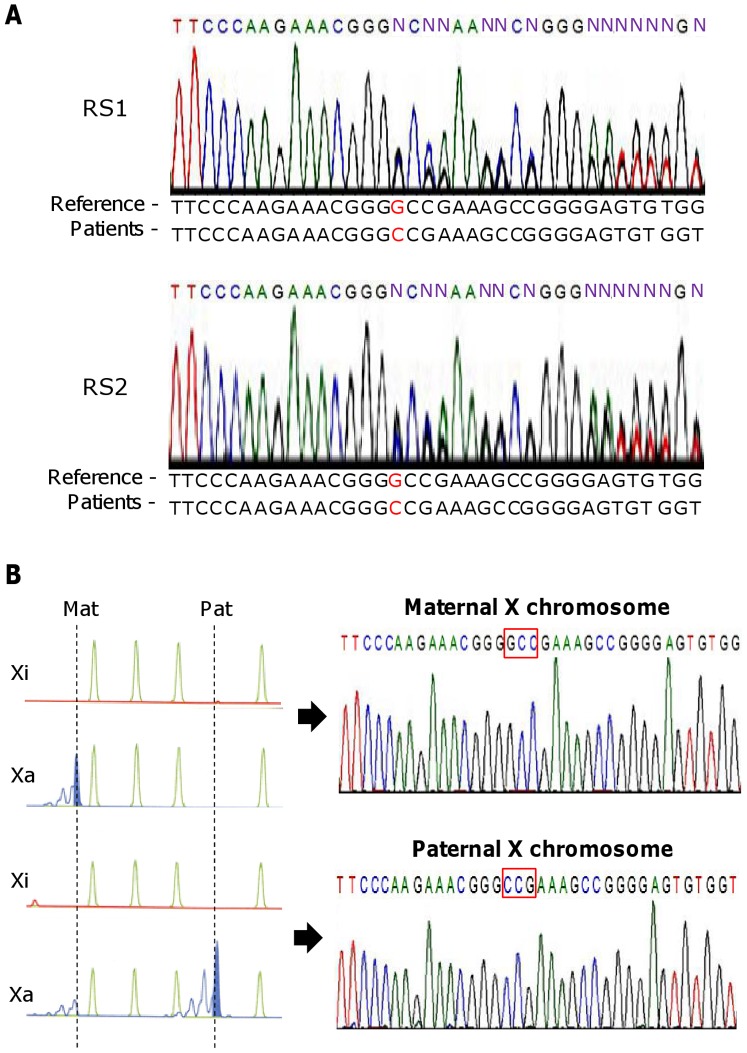
Mutation analyses of the *MECP2* gene in the RTT twins. (A) DNA sequencing demonstrated that both twin 1 (RS1) and twin 2 (RS2) had the same mutation (1 bp “G” deletion in exon 4). (B) Determination of the parental origin of the mutation. (Upper) Sequencing of *MECP2* in a somatic hybrid cell clone carrying an RS1 derived maternal X chromosome. (Lower) Sequencing of *MECP2* in a somatic hybrid cell clone carrying an RS1 derived paternal X chromosome. The parent of origin of the X chromosome was determined by the HUMARA X chromosome inactivation assay.

DNA sequencing analyses demonstrated that both twins had the same frame-shift mutation resulting from a 1 bp deletion (c.806delG, p.Gly269Ala*fs*X288) in exon 4 of *MECP2* in the middle of the transcriptional repression domain (Fig. 1A). This mutation has previously been identified in two other RTT patients [33]. The mutation was not found in either the father or the mother (data not shown), indicating that it is *de novo*.

### Parent-of-origin of the *MECP2* Mutation

To determine whether the *de novo* mutation occurred in the germ cells of the father or the mother, mono-chromosomal hybrid cell lines were established from the twins. We performed quinacrine plus Hoechst 33258 staining and identified several clones from each twin carrying a single paternal or maternal chromosome X (data not shown). The human androgen receptor (HUMARA) assay (Fig. 1B, left), followed by DNA sequencing analyses (Fig. 1B, right), demonstrated that the *MECP2* mutation was paternal in origin. This finding is consistent with previous reports of paternally-derived *MECP2* mutations in Rett syndrome patients [34]–[36].

### XCI Patterns in Various Tissues of the Twins

Rett syndrome is an X-linked dominant disease caused by a *MECP2* mutation. Thus, a favorable XCI pattern is one in which the predominant cell population has the active X with the normal *MECP2* allele. This was the pattern expected of twin 1 (RS1) who had the milder phenotype. The opposite pattern, that is, a predominant cell population with the active X carrying the mutant allele, was expected in the second twin (RS2) who showed a more severe phenotype.

**Figure 2:**
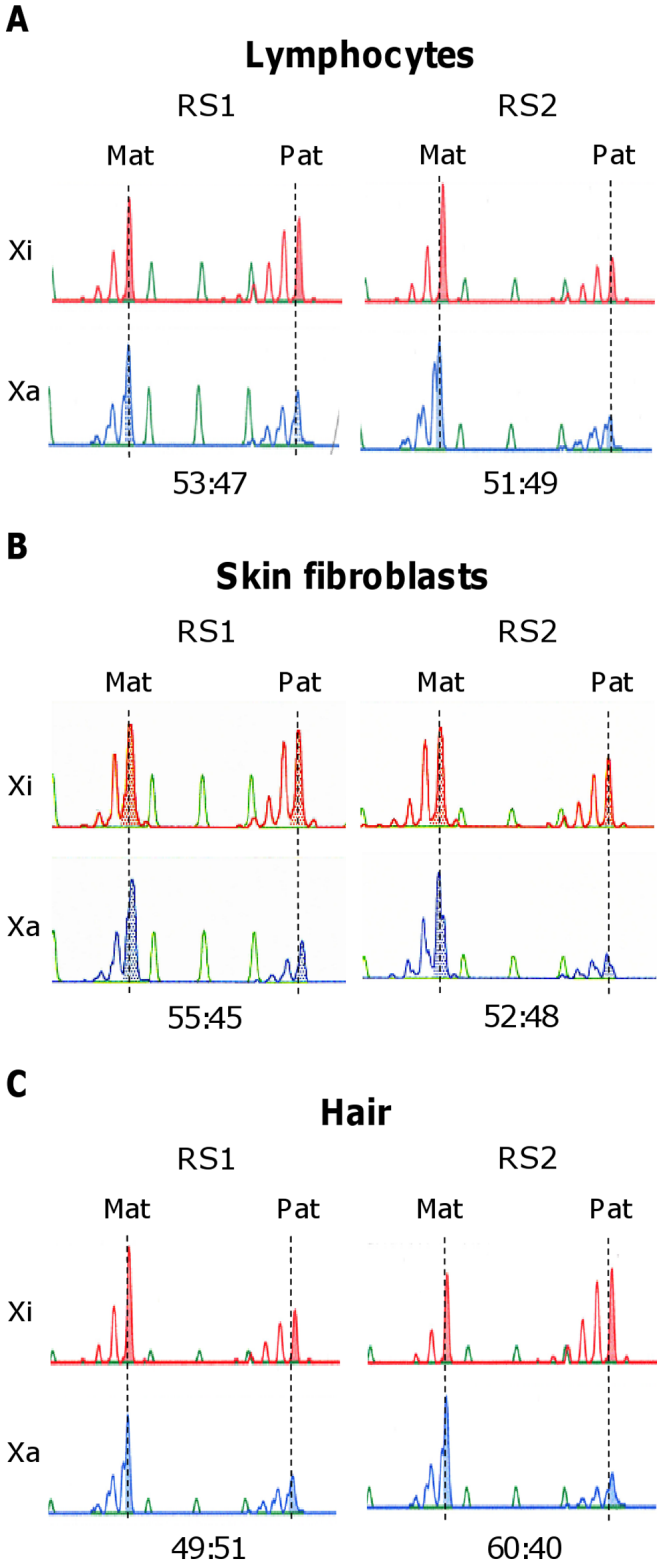
X chromosome inactivation analyses. Xi: X inactivation pattern based on the inactive X chromosome, Xa: X-inactivation pattern based on the active X chromosome. Mat: X chromosome inherited from the mother, Pat: X chromosome inherited from the father. Xi and Xa are differentiated by their methylation status at the androgen receptor gene locus. The maternal X and the paternal X are also differentiated by CAG repeat polymorphism at this locus. The X chromosome inactivation patterns showed no differences between the twins in lymphoblasts (A), skin fibroblasts (B), or hair cells (C).

In this context, we examined the patterns using the HUMARA assay based on methylation-specific PCR [24]. As a result, the patterns in peripheral blood lymphocytes were 53:47 and 47:53 in RS1 and RS2, respectively, for the active normal allele to active mutant allele (Fig. 2A), and the patterns in skin fibroblasts were 55:45 and 52:48 in RS1 and RS2, respectively (Fig. 2B).

Lymphocytes and skin fibroblasts originate from the embryonic mesoderm, whereas the origin of neuronal tissue (the target tissue of this syndrome) is ectodermal. Since XCI patterns vary among tissues of different embryonic origin but are similar within those of the same embryonic origin [37], we analyzed XCI patterns in hair cells, which are of ectodermal origin. This analysis yielded ratios of 49:51 and 60:40 for RS1 and RS2, respectively (Fig. 2C). Overall, the analyses of the various tissues indicated that there was no difference in XCI patterns between the twins.

### SNP Genotyping in the Twins

A total of 24,500,000 chromosomal SNPs were genotyped in the twins using the Illumina Human 610-Quad BeadChips. The overall rate of success for SNP genotyping was 99.99% (24,474,618). In the 24,474,618 SNPs that were successfully genotyped in both twins, there were 19 SNP differences. Each SNP with a difference was either heterozygous in one of the twins and homozygous in the other twin, or homozygous in both twins but with different nucleotides. These putative 19 SNP differences were validated using Solexa whole genome sequencing by HiSeq2000 (Illumina). However, the base sequences of all 19 SNPs were identical in the twins, suggesting typing errors as the source of the 19 SNPs in the Illumina Human 610-Quad BeadChips.

In the Illumina sequencing, the average insert size of each paired-end library was 322 bp (RS1) and 329 bp (RS2) with 100 bp paired-end reads, respectively. After alignment to the human reference genome and removal of PCR duplications, we obtained high-quality nucleotide sequences of 114.9 Gb (40.2× coverage) from RS1 and 103.0 Gb (36.1× coverage) from RS2. The read coverage was 99.9% of the human reference genome (both RS1 and RS2). Comparison of their genomes revealed no specific variants in positions covered with at least 20× depth (at least two reads of each orientation).

### Indel Genotyping in the Twins

Using Solexa whole genome sequencing by HiSeq2000 (Illumina), we also investigated the twins for differences with respect to deletions, insertions, inversions, intra-chromosome translocations and inter-chromosome translocations. We detected one difference, a deletion, between the twins. We attempted to validate this putative deletion by PCR using a primer pair encompassing the affected region. However, the PCR failed to demonstrate the deletion, suggesting that the supposed deletion was a detection error in the Solexa whole genome sequencing (Illumina).

### CNV Detection in the Twins

In addition to the SNP typing probes, 2,419,193 CNV probes in the Illumina Human 610-Quad BeadChips were used to detect CNVs in the twins. With respect to these CNVs, cnvPartition 1.0.2. demonstrated that 2,410,998 were matched between the twins, whereas 8,195 were not matched. Solexa whole genome sequencing was used to validate these putative 8,195 CNV differences; copy number differences were found for 29 CNVs, with RS1 having three copies and RS2 two copies.

A digital PCR assay was used for further validation of these 29 different CNVs; this analysis found no copy number differences between the twins. Overall, therefore, we could not find any genotypic differences between these monozygotic twins.

### DNA Methylation and Expression Differences between the Twins within the *MECP2* Region

**Figure 3:**
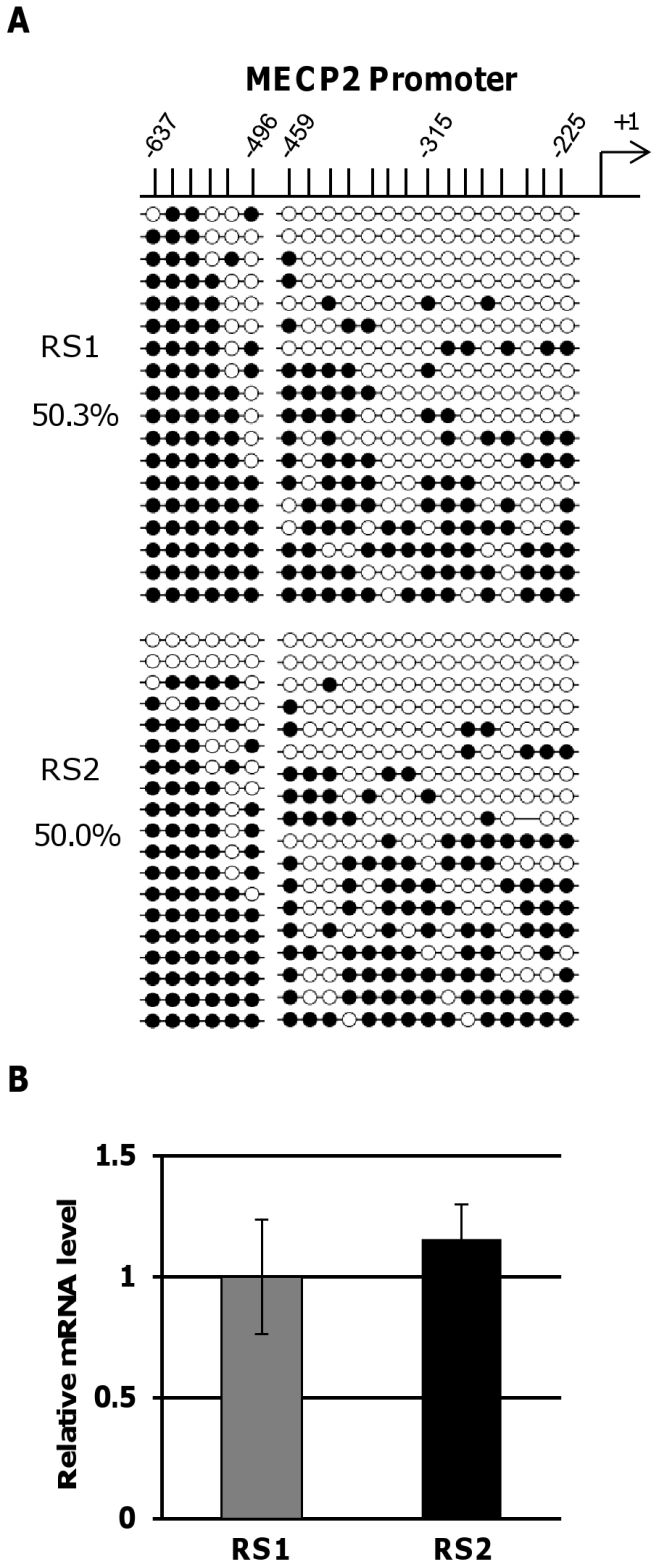
DNA methylation and expression analyses for the *MECP2* gene. (A) DNA methylation profile in the upstream region of *MECP2* by bisulfite sequencing. The upper number indicates the position of the CpG from the transcription start site of *MECP2*. (B) Results of a qRT-PCR assay for MECP2 gene.

It is generally accepted that an unfavorable XCI pattern is associated with a more severe RTT phenotype because of the reduced expression of normal *MECP2* [38]. As genome-wide differences in DNA methylation have also been reported between MZ twins [39], we postulated that *MECP2* expression might also be reduced by hypermethylation of the *MECP2* promoter region in the twin that shows the more severe phenotype. However, bisulfite sequencing did not show any DNA methylation difference in the promoter region of fibroblasts from the twins (RS1, 50.3%; RS2, 50.0%) (Fig. 3A). In agreement with the methylation results, a qRT-PCR assay did not find a significant difference in *MECP2* expression in the fibroblasts of the twins (Fig. 3B).

### Genome-wide Survey of DNA Methylation Difference between the Twins

Although we did not find any DNA methylation differences within the *MECP2* region, it was still possible that such differences were present elsewhere in the genomes of the twins [39], and these differences might alter gene expression patterns to produce the different clinical phenotypes. Therefore, we searched for differentially methylated regions between the twins using an array method that allowed us to determine the DNA methylation status of 450,000 CpG dinucleotide sites in the human genome (mostly located in gene promoter regions) [40].

**Figure 4:**
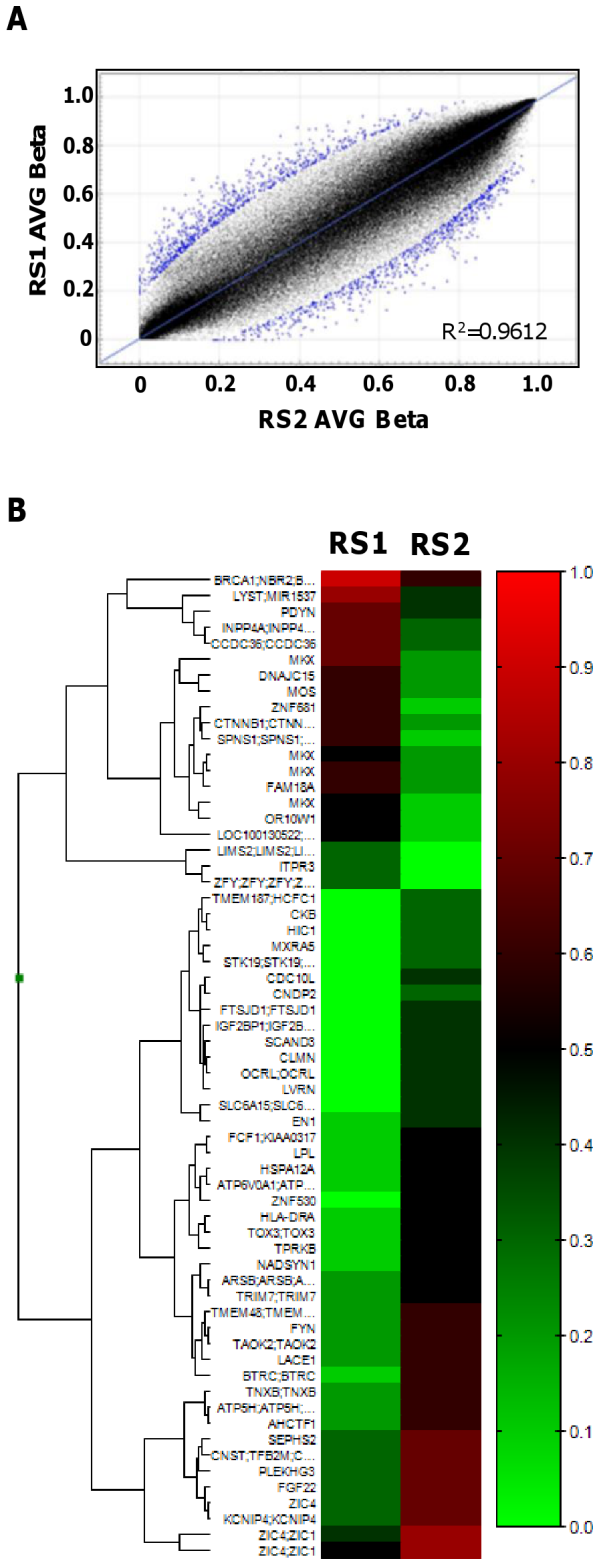
Results of genome-wide analysis of DNA methylation in the twins. (A) Correlation of DNA methylation at the CpG sites analyzed between the twins based on an average (AVG) beta value. (B) Differentially methylated CpG sites between the twins. Red indicates hypermethylation and green indicates hypomethylation.

After normalization and data processing of the array, the full dataset contained 485,577 loci. We found that the methylation levels of these loci mostly matched in the fibroblast samples from RS1 and RS2 (R^2^ = 0.9612) (Fig. 4A). However, 252 loci showed a distinct DNA methylation difference (DiffScore >|80|) between the twins: RS1 (with the milder phenotype) showed higher DNA methylation than RS2 (with the more severe phenotype) at 100 loci; whereas, RS2 showed higher DNA methylation than RS1 at 152 loci (Fig. 4B).

### Validation of DNA methylation and expression status at selected sites

**Figure 5:**
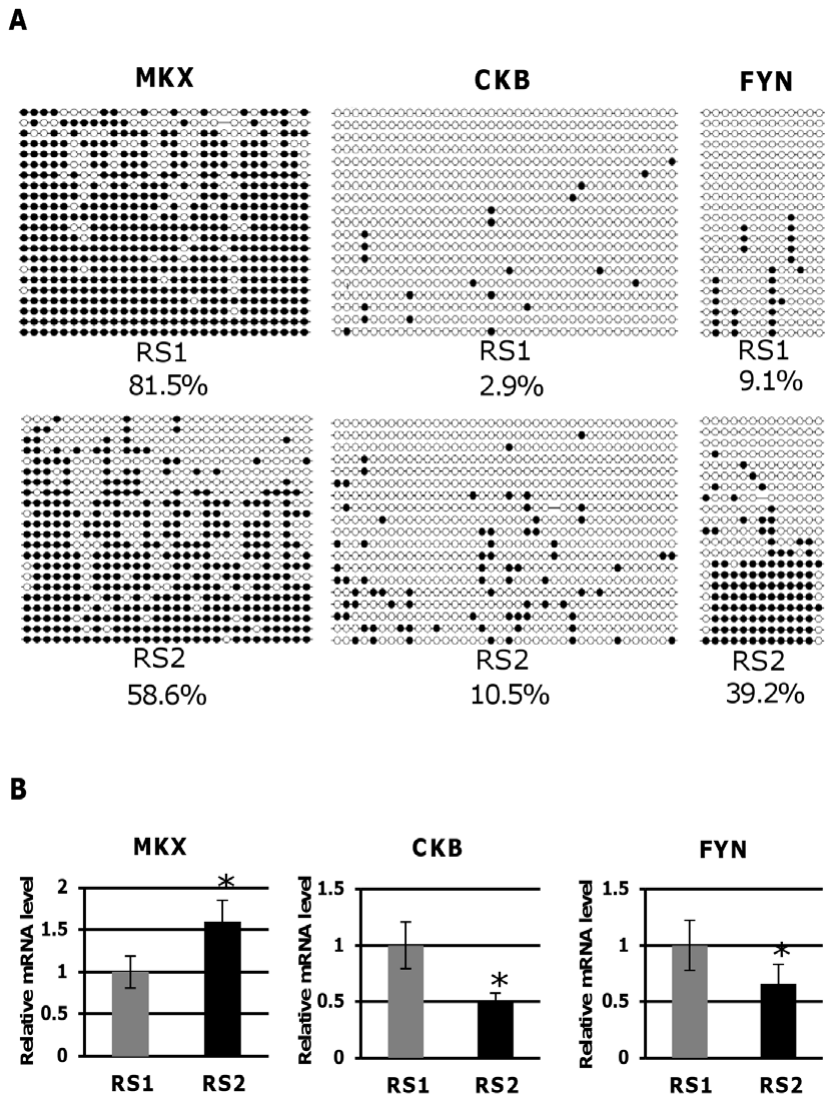
Validation of DNA methylation status and expression difference between the twins. (A) DNA methylation difference between the twins by bisulfite sequencing in the upstream regions of *Mohawk Homeobox* (*MKX*), *Brain-type Creatine Kinase* (*CKB*), and *FYN Tyrosine Kinase Protooncogene* (*FYN*) genes. The proportion (%) of methylated CpG sites is shown. (B) Expression difference between the twins. *p<0.05.

Among the 252 loci identified in the above analysis, we selected 14 that were located upstream of the transcription start site. We performed a bisulfite sequencing analysis to confirm the methylation status at these 14 loci. Significant differences in methylation status between the twins were confirmed for 3 of the 14 loci, namely *Mohawk Homeobox* (*MKX*), *Brain-type Creatine Kinase* (*CKB*), and *FYN Tyrosine Kinase Protooncogene* (*FYN*). The differentially methylated sites are located upstream of the transcription start site of these genes, which might contribute to the clinical differences between the twins. For the upstream region of *MKX*, RS1 showed higher DNA methylation than RS2; however, for the upstream regions of *CKB* and *FYN*, RS2 showed higher DNA methylation levels (Fig. 5A). These results were consistent with those from the genome-wide methylation analysis. As expected, *MKX* expression was lower in RS1 than RS2, and *CKB* and *FYN* expression was higher in RS1 than RS2 (Fig. 5B). These expression patterns were consistent with the methylation status of each gene obtained from the genome-wide and bisulfite sequencing methylation analyses.

## Discussion

In the present study, we examined the genome, epigenome and expression patterns of MZ twins discordant for RTT. We found that (1) the twins shared the same *de novo MECP2* mutation; (2) the *de novo* mutation was of paternal origin (occurred in spermatogenesis); (3) XCI patterns did not differ in various peripheral tissues between the twins; (4) no inter-twin difference was found in whole genome sequences; (5) there were no differences in DNA methylation of the *MECP2* promoter region, nor did *MECP2* expression differ between the twins; (6) the DNA methylation status of a number of loci varied between the twins; (7) this DNA methylation difference was confirmed by the effect on expression of three genes, which may contribute to clinical differences between the twins. These results indicate that epigenetic differences, but not genetic differences, appear to be associated with the discordance between these twins.

Thanks to the advances in next generation sequencing technology, several whole genome sequencing studies have now been conducted to identify differences between MZ twins. Whereas SNP differences have not been reported between MZ twins, CNV differences between MZ twins have been reported [40]–[43]. A possible mechanism for this phenomenon can be suggested from the observation that aphidicolin (an inhibitor of eukaryotic nuclear DNA replication) can induce replication stress in normal human cells leading to a high frequency of copy number changes across the human genome that closely resemble CNVs [44]. Thus, CNV differences between twins might be induced by environmental factors. However, there are also lines of evidence that suggest CNVs do not differ between MZ twins [15], [45], [46]. In our study, candidate CNV loci identified by a combination of next-generation sequencing and a CNV/SNP chip assay could not be validated by a digital droplet-based PCR quantitative assay. Our analysis indicates that the DNA sequences in primary skin fibroblasts are stable post-zygotically. This conclusion is supported by a recent study of induced pluripotent stem cells (iPSCs) using a digital droplet PCR assay; this study reported that somatic reprogramming did not lead to *de novo* CNVs in iPSCs derived from the fibroblasts [29]. Our results also caution that CNV data obtained by next-generation sequencing or a CNV/SNP chip assay should be validated by a quantitative assay.

Initially, DNA methylation analysis was performed at the single gene level by a method based on Southern blotting; subsequently, a methylation-specific PCR assay in combination with sodium-bisulfite treatment of DNA was used [23], [47]. Recently, it has become possible to investigate genome-wide DNA methylation patterns using a method based on a CpG island microarray with sodium-bisulfite treated DNA [48] or with methylated DNA immunoprecipitation (MeDIP) [49]. In this study, we used an assay based on a CpG island microarray with sodium-bisulfite treated DNA. The assay encompassed 450,000 CpG dinucleotides in the human genome, and identified a number of loci with different DNA methylation patterns between the twins. However, the target of this method was mainly gene promoter regions and the coverage was only ∼2% of the human genome. Therefore, it is still possible that there are other regions where DNA methylation differences are more distinct between the twins. Next generation sequencing methods with MeDIP or a new sequencer which allows direct reading of 5-methylcytosine may be able to determine whether this possibility actually occurs. These methods will be useful for ongoing twin cohort studies [50].

In the comprehensive DNA methylation assay, we identified loci with differential methylation in the upstream regions of some genes. We were able to show that the twins showed gene expression differences that were in accord with the methylation patterns of three genes, namely *MKX*, *CKB* and *FYN*.

*MKX* encodes a transcription repressor that is mainly expressed in the embryonic progenitor cell populations of skeletal muscle, tendon, bone and cartilage, and which is an essential factor for musculoskeletal and tendon differentiation [51], [52]. Knockout mice experiments demonstrated that deficiency of this molecule leads to abnormal tendon sheaths and morphological abnormalities in muscle satellite cells. In our study, over-expression due to promoter hypomethylation was observed in the twin with the more severe phenotype (RS2). Although this analysis was confined to fibroblasts and the significance of its over-expression in these cells is not well understood, it is possible that the expression difference was associated with the clinical differences in bones and muscles between the twins, because the more severely affected individual (RS2) had greater muscle weakness than her sibling (RS1).

*CKB* encodes a brain isoform of creatine kinase that plays a crucial role in brain energy homeostasis and an important role in GABA neurons by activating the neuron-specific K^+^-Cl^-^ co-transporter KCC2 [53]. Furthermore, *CKB* is decreased in the brains of patients with neurodegenerative disorders including Alzheimer disease, Huntington disease, and Pick disease [54]–[56]. *CKB* also has an important role in the bone-reabsorption function of osteoclasts [57]. Although down-regulation due to DNA methylation was only confirmed in peripheral tissues, it is intriguing to speculate that the decreased *CKB* expression found in RS2 might contribute to her more severe neurodevelopmental features.

*FYN*, a member of the Src family of kinases associated with neuronal signaling, regulates phosphorylation and trafficking of *N*-methyl-D-aspartate receptors (NMDARs) [58], molecules that are important to synaptic plasticity and learning [59]. *FYN* is also one of the dephosphorylation-target substrates of striatal-enriched protein tyrosine phosphatase (STEP). STEP dysregulation is involved in neuropsychiatric disorders, such as Alzheimer’s disease, schizophrenia, and fragile X syndrome [60]. Therefore, down-regulation of this molecule due to hypermethylation may be associated with the greater mental retardation observed in the more severely affected twin (RS2), although it was obviously not possible to confirm the methylation and expression status in neuronal cells of the twins.

In this study, we mainly targeted upstream promoter regions of genes, because the methylation status of these regions is known to be associated with differences in gene expression patterns. However, a recent study suggested that the methylation status of intragenic (alternative promoter) regions may also be important to the control of gene expression [61]. Therefore, a whole-genome DNA methylation analysis by MeDIP-sequencing will be necessary to determine the influence of possible methylation differences in intragenic regions in the etiology of the discordance between the twins.

DNA methylation studies have been performed in patients with mental diseases, and disease-specific aberrant methylation has been found in neuronal gene regions in these patients [13]. Moreover, it has been reported that there is a correlation between peripheral DNA methylation status and brain function as assessed by positron emission tomography [62]. However, since DNA methylation status varies among tissues, it will definitely be important to determine the specific DNA methylation status of the brains of patients.

A recent development in clinical studies of neurological diseases, including RTT, is to exploit iPSCs in order to understand the consequences of genetic changes in target tissues, such as neuronal cells [63]. Although iPSCs are believed to faithfully recapture the developmental course of the disease of interest, iPSCs cannot maintain stochastic or environmentally-induced alterations in epigenetic status, including DNA methylation, because the epigenetic status is erased during the establishment of iPSC cell lines. Thus, twin-derived-iPSCs will not recapture the original DNA methylation status. It will require further technical breakthroughs, such as development of real-time imaging systems to monitor DNA methylation status in patient brains, to advance our understanding of the role of changes in methylation in the development of neurological diseases.
