# Correction: Comparison of Genomic and Epigenomic Expression in Monozygotic Twins Discordant for Rett Syndrome

**DOI:** 10.1371/journal.pone.0094737

**Published:** 2014-04-09

**Authors:** The PLOS ONE Staff

**Notice of Republication**

This article was republished on March 14, 2014, to remove confidential or copy-righted information. Please download this article again to view the correct version.
